# Over- and under-estimation of COVID-19 deaths

**DOI:** 10.1007/s10654-021-00787-9

**Published:** 2021-07-28

**Authors:** John P. A. Ioannidis

**Affiliations:** 1 Department of Medicine, Stanford University, Stanford, CA USA; 2 Department of Epidemiology and Population Health, Stanford University, Stanford, CA USA; 3 Department of Biomedical Data Science, Stanford University, Stanford, CA USA; 4 Department of Statistics, Stanford University, Stanford, CA USA; 5 Meta-Research Innovation Center At Stanford (METRICS), Stanford University, Stanford, CA USA; 6 Stanford Prevention Research Center, Medical School Office Building, Room X306, 1265 Welch Road, Stanford, CA 94305 USA

**Keywords:** COVID-19, Mortality, Diagnostic testing, Death certificates, Excess deaths

## Abstract

The ratio of COVID-19-attributable deaths versus “true” COVID-19 deaths depends on the synchronicity of the epidemic wave with population mortality; duration of test positivity, diagnostic time window, and testing practices close to and at death; infection prevalence; the extent of diagnosing without testing documentation; and the ratio of overall (all-cause) population mortality rate and infection fatality rate. A nomogram is offered to assess the potential extent of over- and under-counting in different situations. COVID-19 deaths were apparently under-counted early in the pandemic and continue to be under-counted in several countries, especially in Africa, while over-counting probably currently exists for several other countries, especially those with intensive testing and high sensitization and/or incentives for COVID-19 diagnoses. Death attribution in a syndemic like COVID-19 needs great caution. Finally, excess death estimates are subject to substantial annual variability and include also indirect effects of the pandemic and the effects of measures taken.

**Supplementary Information:**

The online version contains supplementary material available at 10.1007/s10654-021-00787-9.

## Introduction

The tragic loss of life during the COVID-19 pandemic must be carefully measured, to illuminate the dynamics of the pandemic and the best use of interventions. Attribution of death typically uses the WHO guidance [1] and various national guidelines. However, there may be large variability across countries and even between different health systems and physicians in the same country in how deaths are attributed. Even before the major reshuffling of death causes due to COVID-19, death certificates were known to be notoriously error-prone [2, 3].

Much debate about COVID-19 death attribution has centered around the use of testing to establish diagnosis. Under-counting of deaths may occur when no testing is done or testing is false-negative; and over-counting may ensue from false-positive testing [4]. Clinically-based attribution of death causes may correct some testing misclassifications—or may add further misclassifications. Eventually, are COVID-19 over-counted or under-counted? Here, a framework is presented to dissect this complex question.

## Determinants of test-attributed COVID-19 deaths

When a proportion P of a population is infected during an epidemic wave, the number of people who die and who will also be positive for the virus either at death or during a diagnostic time window d preceding death is approximated (as explained in Appendix) by:

$${\text{N}}_{{{\text{test}} - {\text{attributed}}}} = {\text{cPSm}}\left( {{\text{t}} + {\text{d}}} \right)$$

where S is the population size, m is the overall population mortality rate per unit time, t is the average time during which testing is positive, and c is a correction factor that reflects synchronicity (the extent to which the epidemic wave is more active when overall population mortality rate is also higher) (Appendix). This formula focuses on the number of patients who die with (not necessarily from) COVID-19. Moreover, this formula would be suitable to inform us about the number of deaths attributed to COVID-19, if everybody who died was tested when there was viral shedding during the t + d period, and the test had perfect properties (perfect sensitivity and specificity) and there was no effort by clinicians to attribute causality beyond COVID-19 testing availability and the results of COVID-19 testing. However, not all people who die are tested around their death or during the diagnostic time window preceding death. Concurrently, some tests are false-positive, clinicians may try to correct some of these false-positives based on the clinical picture and, conversely, certain deaths are also clinically attributed to COVID-19 despite negative test results or without any testing. N_test-attributed_ must be multiplied by an attributed mortality correction factor X to obtain the attributed COVID-19 deaths:

$${\text{N}}_{{{\text{attributed}}}} = {\text{cPSm}}\left( {{\text{t}} + {\text{d}}} \right){\text{X}}$$

## Determinants of the attributed mortality correction factor

The attributed mortality correction factor Χ can be defined by the product of the probability Π of being detected positive around death or during the diagnostic window; the inverse of the positive predictive value (PPV) of the test (which is given by (sensitivity x prevalence)/[(sensitivity × prevalence) + ((1 – specificity) × (1 – prevalence)]); the probability Λ that a clinician will attribute death to the virus in the presence of positive COVID-19 test results; and a factor Φ that reflects how many deaths overall are proclaimed to be COVID-19-related compared with those that are proclaimed to be COVID-19-related in the presence of a positive test. Hence, X is lower when less testing is done. X increases with decreasing positive predictive value (e.g. when testing is done under conditions of low infection prevalence) [4] and with increasing tendency of clinicians to attribute a death to COVID-19 when they have a positive test. If clinicians had perfect perception of the PPV under different circumstances, they could use Λ to counter fairly the impact of the PPV, but this ideal situation is unlikely to happen and therefore X is probably affected substantially. X also increases when clinicians are more eager to proclaim COVID-19 deaths despite negative tests or no testing.

As a simplified illustrative example, if Π = 50% of patients are tested and captured with positive results around the time of death or during the diagnostic window, the test has perfect sensitivity and specificity (thus perfect PPV, regardless of prevalence of the infection), clinicians attribute Λ = 90% of test-positive deaths to COVID-19 and they attribute 60% more deaths to COVID-19 despite negative or no testing than those that they attribute to COVID-19 with positive testing (Φ = 1 + 0.60), then X = 0.50 × 1 × 0.90 x (1 + 0.60) = 0.72. However, given that the test is not perfect, if the prevalence of the infection is very low and the positive predictive value is 0.6 (only 60% of the positive tests truly reflect shedding), then under the same circumstances X would be 0.72/0.6 = 1.2.

There have been many systematic reviews and meta-analyses of the diagnostic performance of testing with RT-PCR, e.g. see references [5–8]. The available data suggest that sensitivity may vary depending on the source of sample and sampling process and specificity may also not be perfect. Positive predictive value would thus largely depend on prevalence, and with low prevalence PPV can also be low [4]. The extent of testing at or close to death and the sensitization of clinicians who attribute death causes have varied extensively over time and across countries and locations. Values of Φ have also varied a lot. The most extreme example of a high Φ value may be Peru where in June 2021 the number of COVID-19 deaths was revised upwards threefold to include deaths with no testing or negative testing [9].

## Ratio of COVID-19-attributed deaths to truly caused COVID-19 deaths

The true number of deaths caused by COVID-19 is N_truly-caused_ = PSF where F is the infection fatality rate. Therefore, the ratio of COVID-19-attributed deaths to truly caused COVID-19 deaths is:

$${\text{R}} = {\text{cPSm}}\left( {{\text{t}} + {\text{d}}} \right){\text{X}}/{\text{PSF}} = {\text{c}}\left( {{\text{t}} + {\text{d}}} \right){\text{Xm}}/{\text{F}}$$

Thus, R is larger when there is stronger synchronicity of the epidemic wave with population mortality; when the test remains positive longer; when the diagnostic time window is set to be longer; when more people are tested close to their death or during the diagnostic time window and/or testing is done in low infection prevalence situations and/or many deaths are coined as COVID-19 without testing documentation; when the population mortality rate is higher; and when the infection fatality rate is lower.

Notably, m and F are not independent. Other things being equal, infection fatality rate is higher in populations with larger shares of elderly and debilitated individuals, and these populations have higher overall mortality rates. Both m and F show strong age-gradients [10]. Both m and F may depend also on various comorbidities and their strength of dependence on different factors may vary, i.e. for some factors m may have a stronger dependence than F does, and the opposite may hold true for other risk factors. Overall, m and F are only modestly correlated, since overall mortality includes deaths from many causes that strike many young people, especially in the developing world, e.g. many African countries have high m despite very young populations [11]. Moreover, different interventions may make m and F even more disjoint. E.g., if a public health or vaccination strategy protects foremost the elderly/vulnerable (“precision shielding”) [12], F may markedly decrease while m is affected far less, thus R may increase substantially. Conversely, if elderly/vulnerable are more frequently infected than the general population (e.g. as seen in massive nursing home outbreaks), R may decrease substantially. Effective treatments or use of detrimental treatments that decrease or increase F, respectively, would also have less impact on overall population mortality rates.

## Changes during the course of the pandemic

The synchronicity correction c probably shows limited variability (Appendix). Also, the average duration of test positivity [13–15] may not have changed much during the pandemic, although it is not precisely known whether it might vary depending on emerging viral variants and on the host population. The diagnostic time window d is typically set at 1 month. However, time from diagnosis to death increases, when mechanical life support is prolonged. With longer d (more commonly in more developed countries), R increases.

Testing volume has increased over time in most locations after little testing was done in the early pandemic. Increased testing would increase R over time other things being equal. However, large variation in testing continues to exist across different locations. Notably, X is specifically related to testing in the diagnostic time window and at death; such testing may not necessarily be strongly correlated with the overall number of tests done in the whole population. Clinically attributing deaths to COVID-19 despite non-congruent test results or lack of testing also varied during the course of the pandemic. Clinicians may have had low suspicion of COVID-19 early on. Conversely, COVID-19 was later seen as a very common condition, not to be missed, and with added incentives to diagnose it; this situation leads to overdiagnosis [16].

Infection fatality rate may decrease over time [17] due to better protection of vulnerable individuals, more effective treatments, better management, and, lately, effective vaccines. If the infection fatality rate decreases steadily and testing remains aggressive (or becomes even more aggressive), an increasingly larger share of COVID-19-attributed deaths may not be causally related to COVID-19 as the pandemic dissipates. Clinicians or auditors of medical records may still dismiss COVID-19 at the cause of death despite test positivity, but typically positive testing places a high burden to code a death as COVID-19.

## Nomogram for attributed over true COVID-19 death counts

**Fig. 1:**
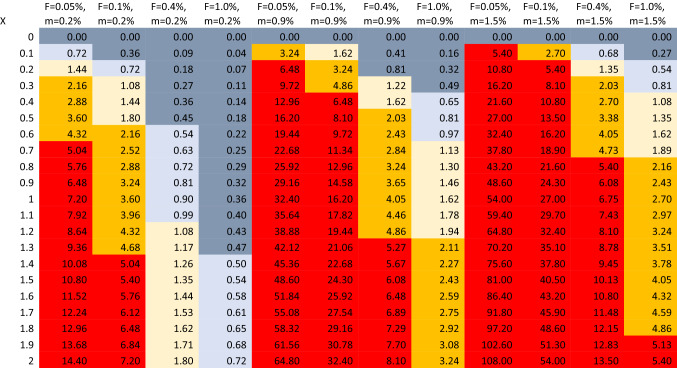
Nomogram of inflation ratio R (test-attributed deaths divided by deaths truly caused by COVID-19) for different combinations of values of X, m, and F. Red color corresponds to r > 5, orange color R = 2–5, yellow color R = 1–2, light blue color R = 0.5–1.0, dark clue color R < 0.5

In the Fig. 1 nomogram, calculations assume c = 1.2, and t + d = 0.15 years (given that typically d = 1 month and considering also typical values for duration of PCR positivity [8–10]. Annual overall population mortality rates vary across different countries [11], from 0.12% in Qatar to 1.54% in Bulgaria. Most countries range between 0.4% and 1.2% (e.g. Iran 0.49%, India 0.73%, USA 0.88%, Sweden 0.92%, United Kingdom 0.94%, Japan 1.06%, Germany 1.13%). Highest values are seen in Eastern European countries and in some African countries. Lowest values cluster in the Arab peninsula. The nomogram considers values of low, moderate and high m (0.2%, 0.9%, and 1.5%, respectively); values of infection fatality rate *F* = 0.05%, 0.1%, 0.4%, and 1%; and values of X from 0 to 2.0. At *R* = 1, the number of recorded COVID-19 deaths equals the number of true COVID-19 deaths (some true COVID-19 deaths may still be missed, but then an equal number of non-COVID-19 deaths are attributed to COVID-19). Values of X > 1 account for imperfect test specificity, and added deaths without test documentation.

For most simulations, *R* exceeds 1 and often reaches high values, i.e. COVID-19-attributed deaths exceed the deaths truly caused by COVID-19 (Fig. 1). Furthermore, some simulations that find *R* < 1 refer to implausible combinations, e.g. very low *m* = 0.2 and high *F* = 1.0 (seroprevalence surveys in Arabian peninsula countries with low m values suggest low F) [18]. Similarly, some extremely high values of R may seem implausible.

*R* < 1 (under-estimated death count) exists primarily when very little testing is done for people who are sick and/or dying and COVID-19 diagnoses are also clinically missed. Most countries may have had low values of X when the pandemic started. Some countries, especially in Africa, may still have low values of X, while most other countries increased X markedly through increased testing and clinical sensitization. Therefore, probably most countries started with underestimates of COVID-19 deaths but as of May 2021 many countries may have overestimates of COVID-19 deaths.

## Illustrative examples for specific countries

USA, UK and Spain have moderate m values (~ 0.9%). If the infection fatality rate in the USA is F = 0.4% [17, 19], then COVID-19 deaths would have been underestimated when X < 0.25, and overestimated for higher X. With X = 0.4, the overestimation would be 1.6-fold; with X = 1.2, it would reach almost fivefold. UK and Spain probably had high infection fatality rate in the first wave [20]: with F = 1.0%, COVID-19 deaths were probably under-estimated. Conversely, the infection fatality rate probably decreased substantially in the second and third waves [20, 21] and thus COVID-19 deaths were probably overestimated. Therefore, while the exact ratio R depends on the specific values that might be assumed for X during the course of the pandemic, it is likely that the cumulative COVID-19 deaths in countries like USA, UK, and Spain and other similar high-income countries have been overestimated. Some of this over-estimation has slowly started to be gradually recognized as of this writing, e.g. in June and July 2021, Alameda and Santa Clara counties in California have reduced their COVID-19 deaths by 25% and 22%, respectively, trying to address overcounting.

Conversely, marked under-estimation of COVID-19 may have occurred in Africa. Data from a study [22] using postmortem nasopharyngeal swabs in Lusaka, Zambia showed that 15.9% (58/364) of deceased in June–September 2020 tested positive for RT-PCR at < 40 cycles and the proportion was 19.2% (70/364) when all RT-PCR positive tests were included regardless of number of cycles needed. 44 of these 70 patients had documented symptoms compatible with COVID-19, but only 6 had also been detected before death. If *R* is estimated as 6/44 = 0.14, it offers a sense of what might be the typical range for the lowest possible values of *R*, given that Zambia had among the lowest testing rates in the world and other features would probably also lead to very low *R*. As of September 16, 2020, only 134,236 tests had been done in Zambia among a population of 18.9 million people (~ 40-fold fewer tests/population than in the USA). African countries that continue to perform limited testing may continue to have underestimates of COVID-19 deaths. However, underestimation may be much more modest now (e.g. *R* = 0.3–0.8), since there is increased sensitization to COVID-19 and increased testing even in Africa (although not everywhere). E.g., Zambia has performed 1.76 million tests as of June 19, 2021 (13-fold more versus September 2020). African countries probably also have very low F, given their young populations and very low rates of obesity (a major risk factor for death with COVID-19), therefore the underestimation problem is probably attenuated.

Among middle-income countries with the highest burdens of COVID-19 fatalities, as of June 19, 2021 the cumulative number of performed tests per 100 people is 25 in Brazil, 28 in India, 5.6 in Mexico, and 41 in Peru (versus 9.4 in Zambia). Therefore, Brazil and India may be facing some under-estimation (but not as major as feared), and India’s under-estimation is further attenuated by a much lower F due to younger population with very low obesity rates compared with Brazil. Under-estimation may be more prominent in Mexico (less testing, probably much higher F than India). Conversely, the recent tripling of attributed COVID-19 deaths in Peru [9] may be highly exaggerated and excess deaths may not reflect directly the virus, but other consequences of the pandemic and of the measures taken, as discussed below.

## Overall mortality rate, age structure and reported COVID-19 mortality rate

**Fig. 2:**
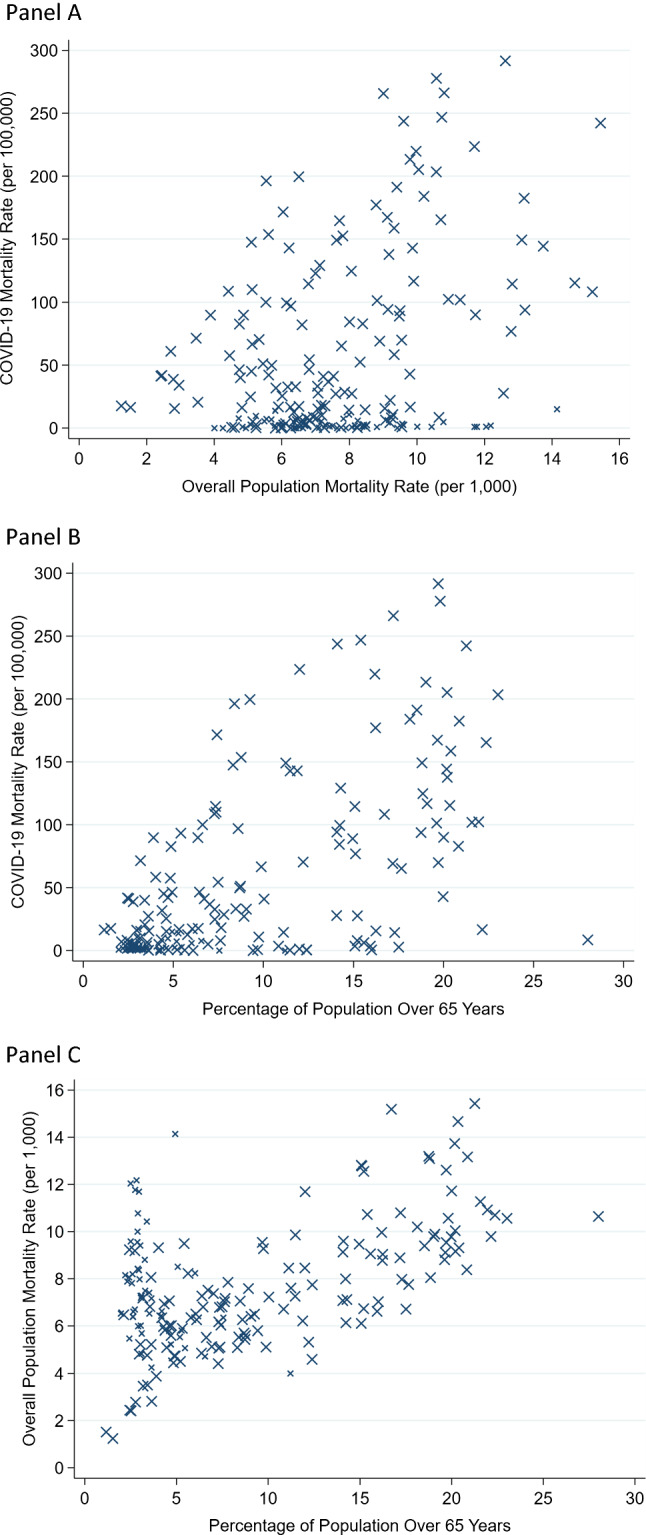
Scatterplots of reported COVID-19 deaths as of May 9, 2021 (in deaths per 100,000 population) against (**a**) overall population mortality rate (annual, per 1000 population) and** b** percentage of population over 65 years. Panel** c** shows a scatterplot of the overall mortality rate against the percentage of population over 65 years. Countries with < 50 tests done per 1000 population during the pandemic and those with no information on number of tests done are shown with smaller markers, since under-counting of deaths is very likely in them. Data for overall mortality rate are from ref. 11, data for COVID-19 mortality are from ref. 21 and both data have been completed also from Ref. 23 and from https://www.indexmundi.com/. Data for age structure are from https://data.worldbank.org/indicator/SP.POP.65UP.TO.ZS. All the data are in the Supplementary data file

The reported COVID-19 mortality rate across countries is modestly correlated with both the overall mortality rate (*r* = 0.38) and with the age structure of the population, in particular the percentage of the population who are over 65 years (*r* = 0.62) (Fig. 2a, b) [11, 21, 23]. Excluding countries where testing for COVID-19 is extremely limited (< 50 tests done per 1,000 people) that under-counting is almost certain, yields correlation coefficients of *r* = 0.48 and *r* = 0.51, respectively. All 11 countries with m < 0.4% have reported < 100 per million COVID-19 deaths as of May 9, 2021. Conversely, 18 of the 23 countries with m > 1.0% have reported > 100 COVID-19 deaths per 100,000 population as of May 9, 2021 (*p* < 0.0001). The 5 exceptions are Serbia (94), Estonia (90) and Russia (77) that are likely to soon exceed 100 reported COVID-19 deaths per 100,000 population (note added in proof: Russia did exceed 100 reported COVID-19 deaths per 100,000 population in mid July, 2021) as well as Belarus (27) and Japan (8). Overall mortality rate and percentage of the population who are over 65 years are also correlated (*r* = 0.54) (Fig. 2c).

The reported COVID-19 mortality rate is a function of the true COVID-19 deaths and of the extent of over- or under-counting conveyed by R and this depends on the ratio m/F. Given the differences in age structure, countries with high m would have higher F than those with low m. However, the steepness of the F differences between these countries would decide if they also have more over-counting or under-counting.

## Challenges in syndemic death counting

COVID-19 is a syndemic [24] where most deaths occur in people with several underlying diseases. Dissecting the relative contribution of each disease/condition to death can be difficult. Careful collection of information on patient characteristics, comorbidities and their severity is essential to get reliable estimates not only for death counts, but also person-years lost and quality-adjusted person-years.

Notably, the infection fatality rate is markedly higher in nursing home residents than in community-dwelling elderly of the same age [25]; and the difference can be extreme, if limited to institutionalized people in palliative care for terminal disease. The extent to which deaths of patients in palliative care with minimum life expectancy are attributed to COVID-19 or not varies across countries and locations. There is large variability across countries on the percentages of people who die at home, at the hospital, or in institutionalized care [26]. These settings may differ in how they pursue diagnosis (or over-diagnosis) of COVID-19 as cause of death. Countries also vary vastly in palliative care availability and organization [27]. One may question whether COVID-19 should ever be listed as primary cause of death in patients with known terminal disease. Paradoxically, countries with better organized health systems and palliative care may report more COVID-19 deaths, especially if they attribute deaths to COVID-19 among patients with known terminal disease. Regardless, person-year calculations would be less biased, if this background information becomes available.

## Validating over- and under-estimation of COVID-19 deaths

Mistrusting reported COVID-19 death counts, several analysts focus on excess death assessments [28]. Excess deaths however have substantial natural annual fluctuation. E.g., Australia and Thailand saw 7% increases in deaths in 2019 versus 2018. Moreover, excess deaths in 2020–2021reflect both COVID-19 and several death causes that possibly increased or decreased during the pandemic. Death certificates have always been inaccurate [2, 3], but COVID-19 maximizes the challenge of prioritizing multiple comorbidities. In the USA, chain-of-event and contributing factor information in COVID-19 deaths seem congruent in most COVID-19 death certificates [29]. However, congruence does not prove accuracy. In many other countries, death certificates are even more unreliable [30]. Some financial incentives [30] may promote coding for COVID-19. The unavoidable alert that a lethal infectious disease is circulating may also affect death cause attribution.

Excess deaths should be scrutinized for death causes accentuated by the pandemic versus by measures taken against the pandemic, e.g. deaths due to disruption of health care, opioid overdoses, suicides, diseases of despair, starvation, tuberculosis, and more [31–34]. Meticulous audit of medical records may offer insights, but even these records may be erratic. Many medical problems mentioned in free text are not entered in the electronic records’ problem list of COVID-19 patients [35]. Autopsies also find many more problems than are otherwise reported [36]. However, autopsies are exceedingly rare [37].

Overall, given these difficulties, equating excess mortality to COVID-19 itself is probably naïve and flawed. Preliminarily, excess mortality in 2020–2021 has been substantially higher than reported COVID-19 deaths in several countries in Eastern Europe, Africa, Mexico, and India [38]. However, the challenge is to disentangle the contributions of COVID-19 itself versus iatrogenic causes (e.g. use of harmful treatments like hydroxychloroquine [39] and inappropriate mechanical ventilation [40]), overwhelmed health systems due to the pandemic versus disrupted health systems due to aggressive, panic-driven measures, and other causes. Even before the pandemic, an estimated 5 million deaths annually worldwide were due to low-quality healthcare [41]. The pandemic and the response to it probably created further challenges, especially for brittle health systems and brittle societies.

### Electronic supplementary material

Below is the link to the electronic supplementary material.

Supplementary file1 (XLSX 18 kb)

## Data Availability

All data used for the analysis are reported in the supplementary file.

## Appendix: Estimating test-attributed COVID-19 deaths

Let P(i) be the probability of being infected during an epidemic wave at any given time point i, P the total cumulative probability of being infected during the epidemic wave, T the total duration of the epidemic wave, t the time period during which a person infected with the virus would test positive, d the diagnostic time window used in death attribution (a death is attributed to the virus if it occurs within d time of a positive test), m(i) the population mortality rate (death from any cause) per unit time at time (i), m the average population mortality rate (death from any cause) per unit time, and S the size of the population of interest.

Initially, one may consider the simplified version where P(i) and m(i) remain steady during the epidemic wave and ask what would happen if one were to test all people at the time of their death. Then the probability of testing positive for the virus at the time of death (for any death and for any cause thereof) during the T + t period is given by

1 $${\text{D}} = {\text{Pt}}/\left( {{\text{T}} + {\text{t}}} \right)$$

For example, if P = 60% of the population is infected, t = 0.07 years, and T + t = 1 year, then D = 0.6 × 0.07/1 = 0.042, i.e. 4.2% of people dying will test positive for the virus, if they happen to be tested at the time of their death, regardless of whether the virus is causally related to the death or is an innocent bystander. The equation is assuming t is substantially shorter than T so that the effect of the initial phase where no full t length “look back” is possible, can be neglected.

One may then ask what would happen if all people were tested continuously during the epidemic wave, not only at the time of their death. Then, the probability of testing positive for the virus either at the time of death or at any time point during the diagnostic time window that would lead to an attribution of the death to the virus is given by

2 $${\text{D}} = {\text{P}}\left( {{\text{t}} + {\text{d}}} \right)/\left( {{\text{T}} + {\text{t}}} \right)$$

For example, if P = 60% of the population is infected, t = 0.07 year, T + t = 1 year, and a death is attributed to the virus if it occurs within d = 0.08 year, then D = 0.6 × (0.07 +  0.08)/1 = 0.09, i.e. 9% of people dying of any cause will be attributed to the virus, if the attribution is done based on the testing alone and without considering any other (e.g. clinical/pathology) information. Then the total number of deaths attributed to the virus during the period T + t with be

3 $${\text{N}}_{{{\text{test}} - {\text{attributed}}}} = {\text{DSm}}\left( {{\text{T}} + {\text{t}}} \right) = \left[ {{\text{P}}\left( {{\text{t}} + {\text{d}}} \right)/\left( {{\text{T}} + {\text{t}}} \right)} \right]{\text{Sm}}\left( {{\text{T}} + {\text{t}}} \right) = {\text{P}}\left( {{\text{t}} + {\text{d}}} \right){\text{Sm}}$$

Moreover, in real circumstances the probability of being infected during the epidemic wave is not steady over time and even the population mortality rate varies over time, e.g. due to seasonality or excess deaths imposed from various causes. Therefore, while (t + d)S can still be considered a constant (t and d are fixed/defined and the population S does not change substantially over time), the product Pm in (Eq. 3) would be more properly replaced by the integral $\int {P(i)m(i)di}$ for values of i from 0 to t + T. This integral is larger than Pm when P(i) and m(i) are synchronized in their variation (P(i) is higher when m(i) is higher) and it is smaller than Pm when P(i) and m(i) are desynchronized in their variation (P(i) is higher when m(i) is lower). It is far more likely that P(i) and m(i) would be synchronized, because infections are more common in winter months, when there is also higher mortality rate in the population. Therefore, one may increase Pm by multiplying with a correction factor c to capture more properly the integral of $\int {P(i)m(i)di}$.

The likely values of c are close to 1. For example, let us consider an annual mortality wave m(i) described for parsimony by a sine function with three scenarios where the peak is (a) 25%, (b) 33.3%, or (c) 50% above the mean (and the trough is correspondingly 25%, 33.3% or 50% below the mean). Illustratively, in scenario (a), mortality may be on the annual average levels on April 15 and October 15, 25% higher than the annual average on January 15, and 25% lower than the annual average on July 15. Let us also consider synchronized P(i) variation with P(i) reaching double the mean at the peak of the mortality wave and reaching 0 at the trough of the mortality wave. E.g. in scenario (a), P(i) reaches its peak on January 15, it is 0 on July 15, and it has values on April 15 and on October 15 that are half the peak value of January 15. Then, c is 1.12, 1.16, and 1.25 in these three scenarios, respectively, depending on how peaked the m(i) variation is. With this correction, (Eq. 3) becomes:

4 $${\text{N}}_{{{\text{test}} - {\text{attributed}}}} = {\text{cP}}\left( {{\text{t}} + {\text{d}}} \right){\text{Sm}}$$
